# Supplementary material for: Bedside Hyperspectral Imaging and Organ Dysfunction Severity in Critically Ill COVID-19 Patients—A Prospective, Monocentric Observational Study
Source: Bioengineering (Basel). 2023 Oct 6;10(10):1167. doi: 10.3390/bioengineering10101167 (PMC10604239; doi:10.3390/bioengineering10101167)
Supplement: Supplementary file 1 [file bioengineering-10-01167-s001.zip › bioengineering-2518678-supplementary.pdf]

bioengineering

MDPI

Supplementary Materials

Bedside Hyperspectral Imaging and Organ Dysfunction Severity in Critically Ill COVID-19 Patients—A Prospective, Monocentric Observational Study

Henning Kuhlmann [1], Lena Garczarek [1], David Künne [1], Kevin Pattberg [1], Annabell Skarabis [1], Mirjam Frank [2], Börge Schmidt [2], Sven Arends [1], Frank Herbstreit [1], Thorsten Brenner [1], Karsten Schmidt [1,†] and Florian Espeter [1,*,†]

[1] Department of Anesthesiology and Intensive Care Medicine, University Hospital Essen, University Duisburg-Essen, 45147 Essen, Germany
[2] Institute for Medical Informatics, Biometry and Epidemiology, University Hospital Essen, University Duisburg-Essen, 45147 Essen, Germany
* Correspondence: florian.espeter@uk-essen.de; Tel.: +49-201-723-84485
† These authors contributed equally to this work.

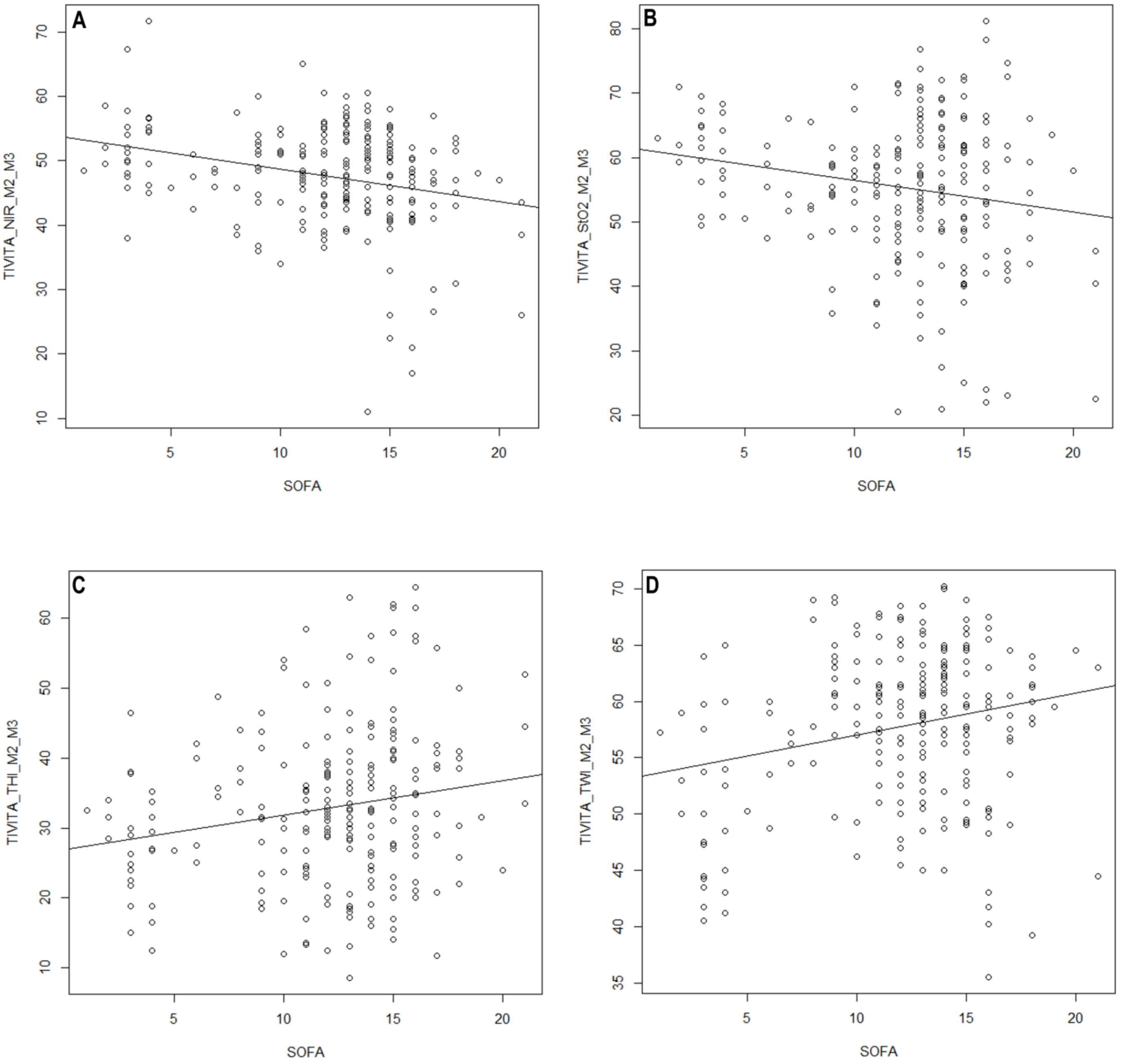

Figure S1. Scatter plots illustrating the association of NPI (**A**), StO2 (**B**), THI (**C**), and TWI (**D**) with SOFA score including all examination time points (day 0, day, 1, day 2, day 3, and day 7).

Table S1. Characteristics of critically ill COVID-19 patients (Espeter et al., 2022) [14]. * Median (inter-quartile range). CCI—Charlson Comorbidity Index; BMI—Body mass index; vvEVMO—veno-venous extracorporeal membrane oxygenation; SOFA—the sequential organ failure assessment score.

**Characteristics of critically ill COVID-19 patients.**	
number of patients *(n)*	52
age (years) *	59 (51–66)
Male *	36 (69%)
clinical data	
CCI *	2 (2–3)
BMI (kg/m²) *	31 (28–36)
at ICU admission	
spontaneous breathing, oxygen dependent	6 (11.5%)
ventilated	46 (88.5%)
non-invasive	6 (11.5%)
invasive	40 (77%)
vvECMO during ICU stay	40 (77%)
initiation at referring ICU	25 (48%)
initiation after ICU admission	15 (28%)
outcome	
28-day survivors	19 (36%)
